# Turmeric: from spice to cure. A review of the anti-cancer, radioprotective and anti-inflammatory effects of turmeric sourced compounds

**DOI:** 10.3389/fnut.2024.1399888

**Published:** 2024-05-28

**Authors:** Mihai Cozmin, Ionut Iulian Lungu, Cristian Gutu, Alina Stefanache, Letitia Doina Duceac, Bogdan Dorin Șoltuzu, Daniela Damir, Gabriela Calin, Elena Roxana Bogdan Goroftei, Carmen Grierosu, Monica Boev

**Affiliations:** ^1^"Apollonia” University of Iasi, Faculty of Dental Medicine, Iași, Romania; ^2^"Grigore T. Popa" University of Medicine and Pharmacy, Iași, Romania; ^3^University Dunarea de Jos Faculty of Medicine and Pharmacy, Galați, Romania; ^4^"Dr. Aristide Serfioti” Military Emergency Clinical Hospital, Galați, Romania; ^5^Prof. Dr. Nicolae Oblu” Neurosurg Hospital Iasi, 2 Ateneului, Iasi, Romania; ^6^Sf. Ioan Emergency Clinical Hospital for Children, 2 Gheorghe Asachi Str., Galați, Romania; ^7^Research Centre in the Medical-Pharmaceutical Field, “Dunarea de Jos” University of Galati, Galați, Romania

**Keywords:** turmeric, chemical composition, antioxidant properties, antioxidant, radioprotectant

## Abstract

Turmeric (*Curcuma longa*) has been extensively studied for its diverse pharmacological properties, including its potential role as an anticancer agent, antioxidant, and radioprotector. This review provides an overview of the chemical composition of turmeric, focusing on its main bioactive compounds, such as curcuminoids and volatile oils. Curcumin, the most abundant curcuminoid in turmeric, has been widely investigated for its various biological activities, including anti-inflammatory, antioxidant, and anticancer effects. Numerous *in vitro* and *in vivo* studies have demonstrated the ability of curcumin to modulate multiple signaling pathways involved in carcinogenesis, leading to inhibition of cancer cell proliferation, induction of apoptosis, and suppression of metastasis. Furthermore, curcumin has shown promising potential as a radioprotective agent by mitigating radiation-induced oxidative stress and DNA damage. Additionally, turmeric extracts containing curcuminoids have been reported to exhibit potent antioxidant activity, scavenging free radicals and protecting cells from oxidative damage. The multifaceted pharmacological properties of turmeric make it a promising candidate for the development of novel therapeutic strategies for cancer prevention and treatment, as well as for the management of oxidative stress-related disorders. However, further research is warranted to elucidate the underlying mechanisms of action and to evaluate the clinical efficacy and safety of turmeric and its bioactive constituents in cancer therapy and radioprotection. This review consolidates the most recent relevant data on turmeric’s chemical composition and its therapeutic applications, providing a comprehensive overview of its potential in cancer prevention and treatment, as well as in radioprotection.

## 1 Introduction

**Figure 1:**
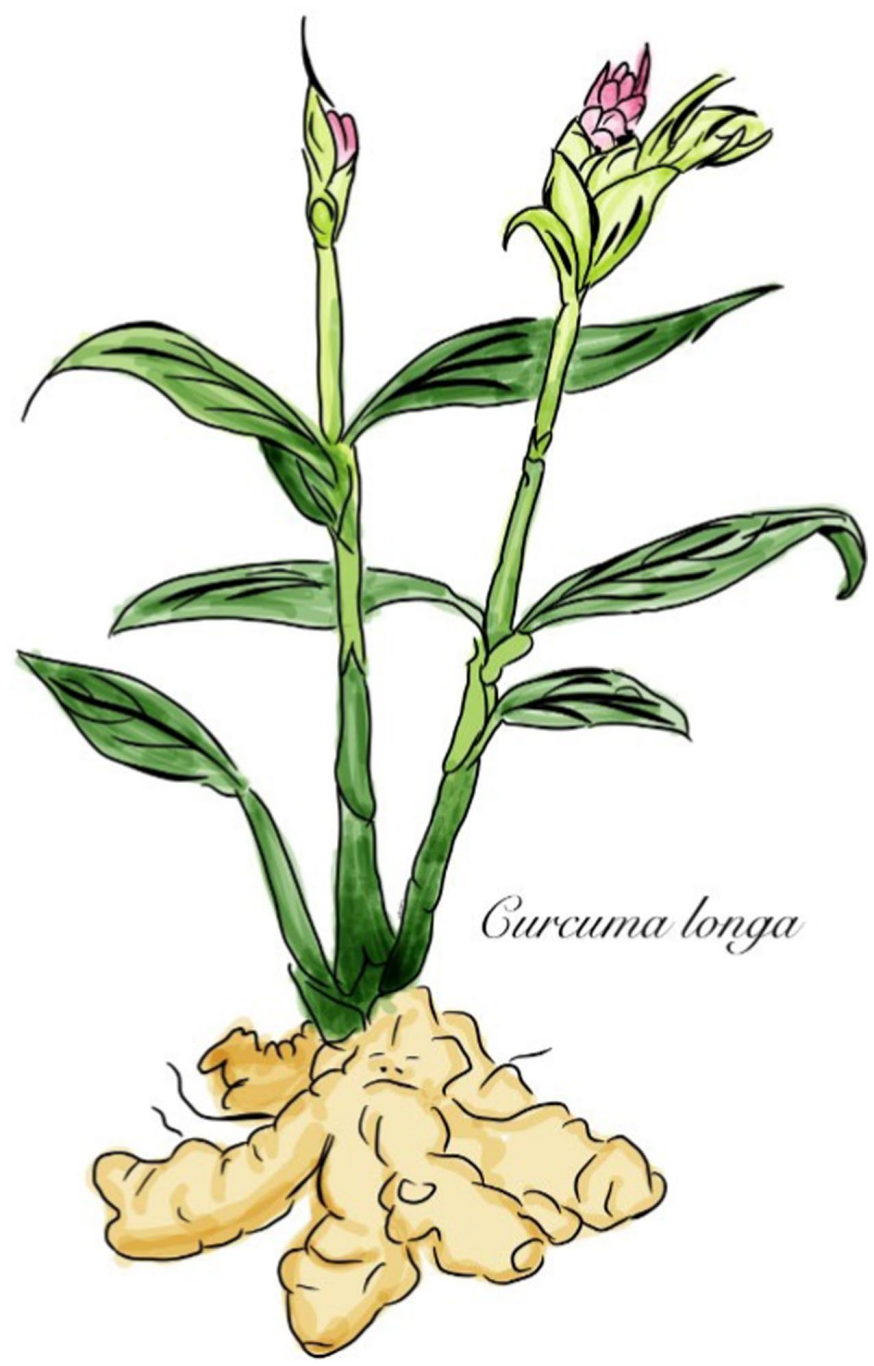
The turmeric plant.

Turmeric (*Curcuma longa*) (Figure 1), originating from India, is a curry spice that has garnered significant attention in recent decades due to its composition of bioactive curcuminoids—curcumin, demethoxycurcumin, and bisdemethoxycurcumin.

**Figure 2:**
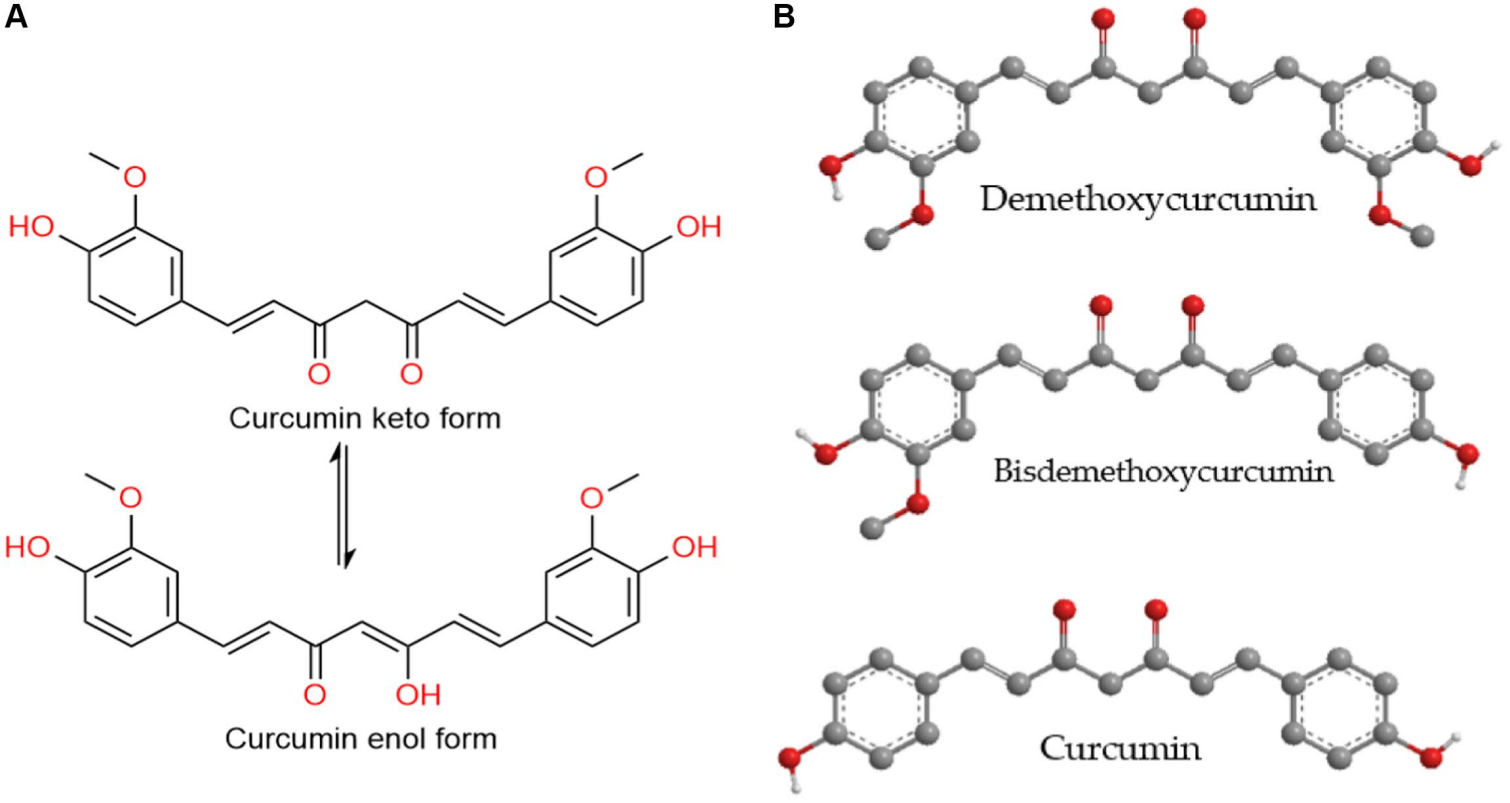
**(A)** The tautomerization of the curcumin molecule. **(B)** Curcuminoids – the main yellow pigments found in turmeric.

Curcumin (Figure 2), known chemically as 1,7-bis-(4-hydroxy-3-methoxyphenyl)-hepta-1,6-diene-3,5-dione, is a lipophilic polyphenol believed to exhibit anticancer, antibiotic, anti-inflammatory, and anti-aging properties, as indicated by various *in vitro*, *in vivo* studies, and clinical trials. Despite its potential, the therapeutic application of curcumin is hindered by challenges such as poor aqueous solubility, limited bioavailability, and unfavorable pharmacokinetic profiles. To overcome these issues, numerous formulations of curcumin have been developed (1, 2).

However, suboptimal sample preparation and analysis methodologies often impede accurate assessments of bioactivities and clinical efficacy. This review provides a summary of recent research on the biological, pharmaceutical, and analytical aspects of curcumin, covering various formulation techniques and discussing associated clinical trials and *in vivo* outcomes.

Turmeric, a member of the *Zingiberaceae* family, is a perennial plant that reaches a height of up to one meter, featuring oblong or cylindrical rhizomes. Externally, these rhizomes are brown and include an egg-shaped primary rhizome known as the “tuber” and multiple branched secondary rhizomes referred to as the “rhizome.” Internally, the rhizomes exhibit colors ranging from yellow to yellow-orange, attributed to pigments called curcuminoids, with diverse pharmacological activities (3–5).

Chemically, curcuminoids, specifically diarylheptanoids, consist of two aryl groups connected by a chain with seven carbons. Among these, curcumin (diferuloylmethane) stands out as the most significant bioactive curcuminoid, alongside others like desmethoxycurcumin and bisdesmethoxycurcumin found in turmeric rhizomes (6).

Extensive research, encompassing preliminary, preclinical, and clinical studies, underscores the pharmacological significance of curcuminoids, the yellow pigment in turmeric. Its versatile properties include anti-inflammatory, immunomodulatory, antioxidant, hypolipidaemic, antimicrobial, anticarcinogenic, antitumor, radioprotective, neuroprotective, hepato-protective, nephroprotective, cardio-protective, and vasoprotective activities (7, 8). Curcumin’s impact extends to various biochemical pathways, influencing molecular targets such as cytokines, transcription factors, kinases, growth factors, and microRNAs (9).

Turmeric, also known as Indian saffron, boasts a rich history of use as an herbal medicine, spice, and coloring agent. Records dating back to 600 BC in an Assyrian herbal book, references by the renowned Greek physician Dioscorides, and mentions in Islamic traditional medicine (ITM) contribute to its historical significance. Turmeric is integral to Chinese traditional medicine (TCM), Ayurveda, and various folk medicines worldwide, with traditional uses ranging from topical treatment for skin disorders to internal remedies for poor digestion and liver function. Recognizing the valuable insights from traditional medicine in guiding natural product-based drug discovery, researchers explore the medicinal applications of turmeric across different traditional systems and investigate the modern pharmacological activities of curcumin, bridging the knowledge from ancient practices to current clinical trials (10, 11).

## 2 Methods

Comprehensive literature searches were conducted across various databases, including Pubmed, Scifinder, ScienceDirect, Medline, Embase, Google Scholar, and Web of Science. The key terms employed for the search encompassed topics such as turmeric, *Curcuma longa*, curcuminoids, curcumin, bioavailability, bioactive compounds, pharmacokinetic, pharmacological effects. Additionally, a thorough examination of articles published in peer-reviewed journals was performed through a library search.

## 3 Biological activities

**Figure 3:**
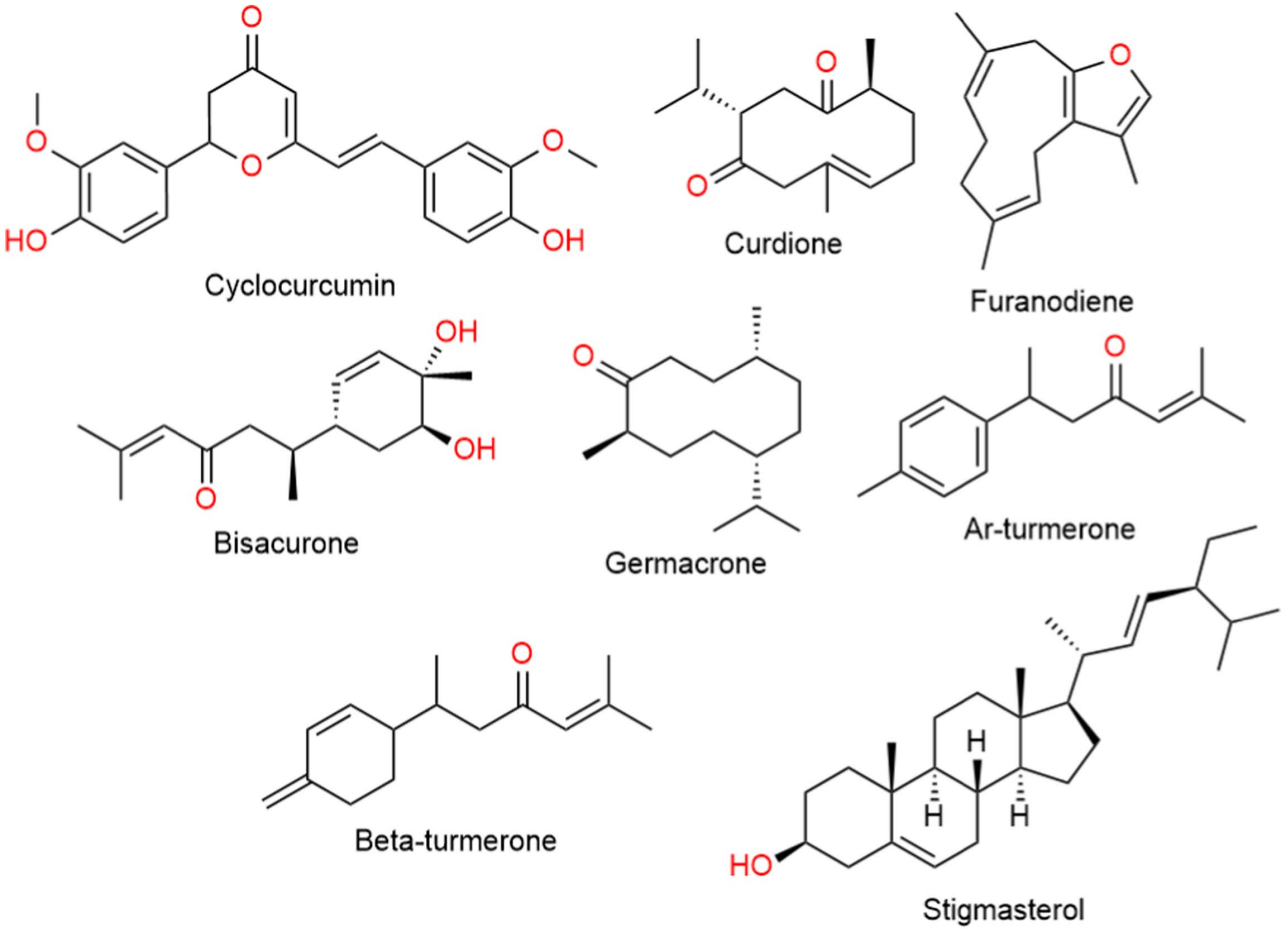
Chemical structure of the common components of turmeric, other than curcumin.

Turmeric displays a rich chemical diversity, with around 235 compounds identified so far, predominantly comprising phenolic compounds and terpenoids (Figure 3). The non-curcumin compounds exhibit diverse chemical structures, including 22 diarylheptanoids, diarylp entanoids, 8 phenylpropenes, various phenolic compounds, 68 monoterpenes, 109 sesquiterpenes, 5 diterpenes, 3 triterpenoids, 4 sterols, 2 alkaloids, and 14 other compounds (12, 13).

**Figure 4:**
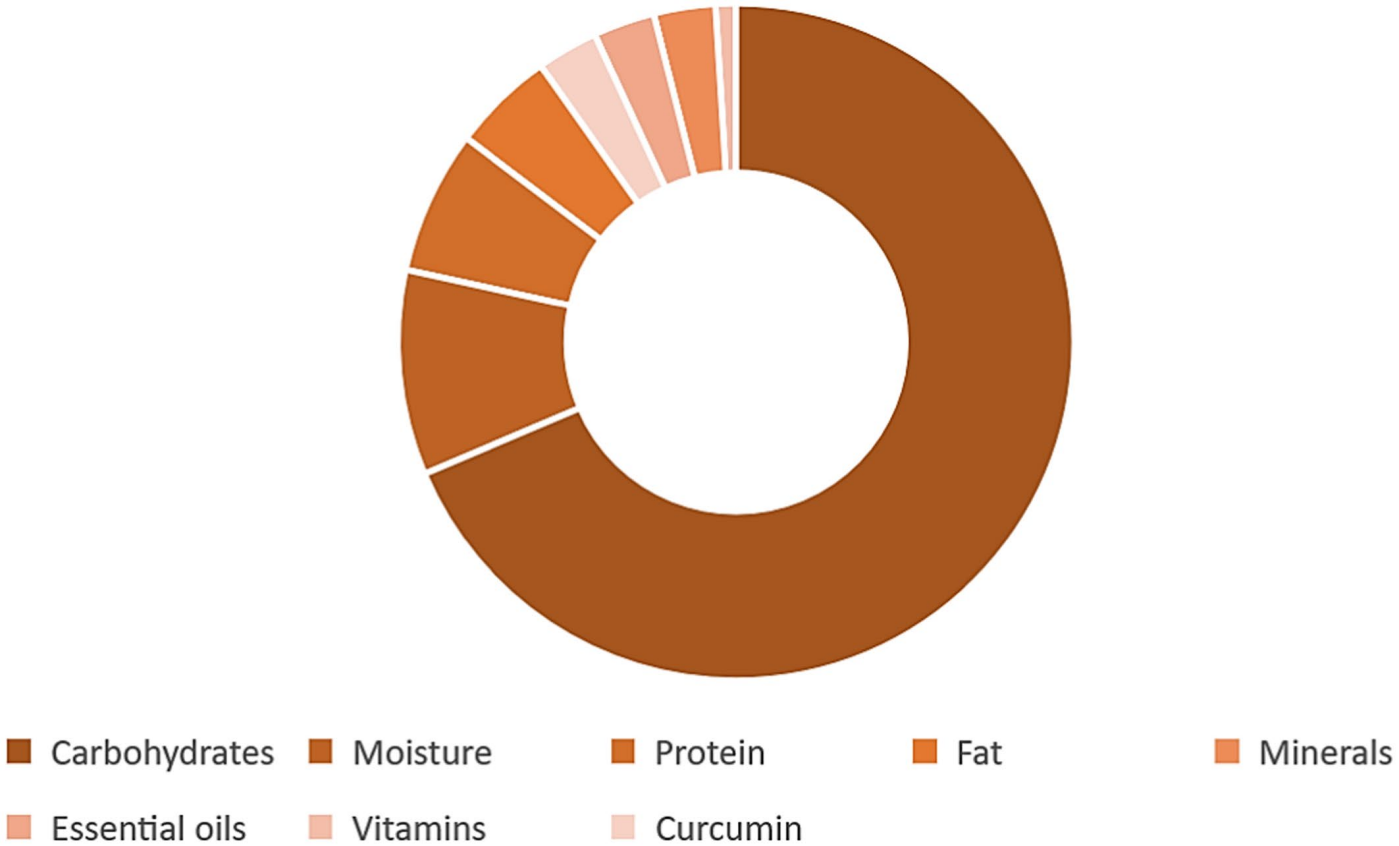
The chemical profile of the turmeric plant.

Turmeric is a plant with a diverse chemical profile (Figure 4 and Table 1), its extracts are obtained using ethanol, methanol, water, or ethyl acetate, and they are both water-soluble and water-insoluble. The water-insoluble fraction comprises turmeric oil and polyphenols, mainly diarylheptanoids (curcuminoids), with curcumin constituting 80%, demethoxycurcumin 18%, and bisdemethoxycurcumin 2%. While 70% ethanol is the preferred solvent for extracting curcuminoids from turmeric, hydrodistillation followed by hexane extraction is the chosen method for separating essential oils. Curcumin, demethoxycurcumin, and bisdemethoxycurcumin collectively may make up over 30% of the ethanol extract of turmeric. Additionally, a distinctive component exclusive to *C. longa* is cyclocurcumin (15, 16).

**Table 1:** The percentage by weight of the compounds found in the turmeric plant (14).

Constituent	Percentage by weight (%)
Curcuminoids	1–6
Volatile oils	3–7
Fiber	2–7
Mineral matter	3–7
Protein	6–8
Fat	5–10
Moisture	6–13
Carbohydrates	60–70

### 3.1 Anti-cancer effects

Curcumin the primary constituents of turmeric has demonstrated efficacy across various stages of cancer progression, exerting inhibitory effects on the transformation, initiation, development, and invasion of tumors, as well as angiogenesis and metastasis. It has been identified as a suppressor of tumor cell growth through modulation of key cellular pathways, including the cell proliferation pathway involving cyclin D1 and c-myc, the cell survival pathway targeting Bcl-2, Bcl-xL, cFLIP, XIAP, and cIAP1, the caspase activation pathway encompassing caspase-8, caspase-3, and caspase-9, the tumor suppressor pathway involving p53 and p21, the death receptor pathway through DR4 and DR5, and various cell signaling pathways, including protein kinase pathways such as c-Jun N-terminal kinases (JNK), protein kinase B (PKB or Akt), and 5′ adenosine monophosphate-activated protein kinase (AMPK) (17).

**Figure 5:**
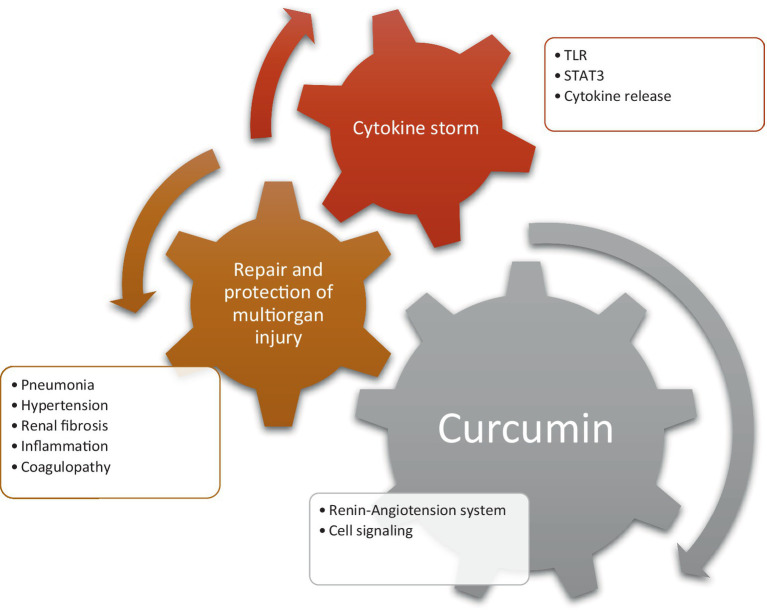
The biological activity of curcumin on different ailments.

Curcuminoids exhibit a diverse range of biological activities (Figure 5). In the context of MCF-7 human breast tumor cells, the impact of curcuminoids and cyclocurcumin was investigated. DMC displayed superior inhibitory effects compared to CUR and BDMC, attributed to the presence of phenolic hydroxyl groups, methoxyl groups, and the diketone moiety. Notably, cyclocurcumin did not influence MCF-7 cell proliferation, indicating that the diketone system within curcuminoids likely contributes to their antiproliferative effects (18).

Semsri et al. explored the influence of pure CUR on the expression of the Wilm’s tumor 1 (WT1) gene in leukemic K562 cell lines. The study revealed that CUR’s effects were mediated through PKCa signaling upstream of the WT1 transcription factor’s auto-regulatory function. Pure CUR impacted WT1 protein-promoter binding, reduced WT1-mRNA levels, and decreased protein levels in K562 cells, contributing to its anti-proliferative effects. This suggests the potential therapeutic utility of CUR in the development of approaches for treating leukemia (19).

**Figure 6:**
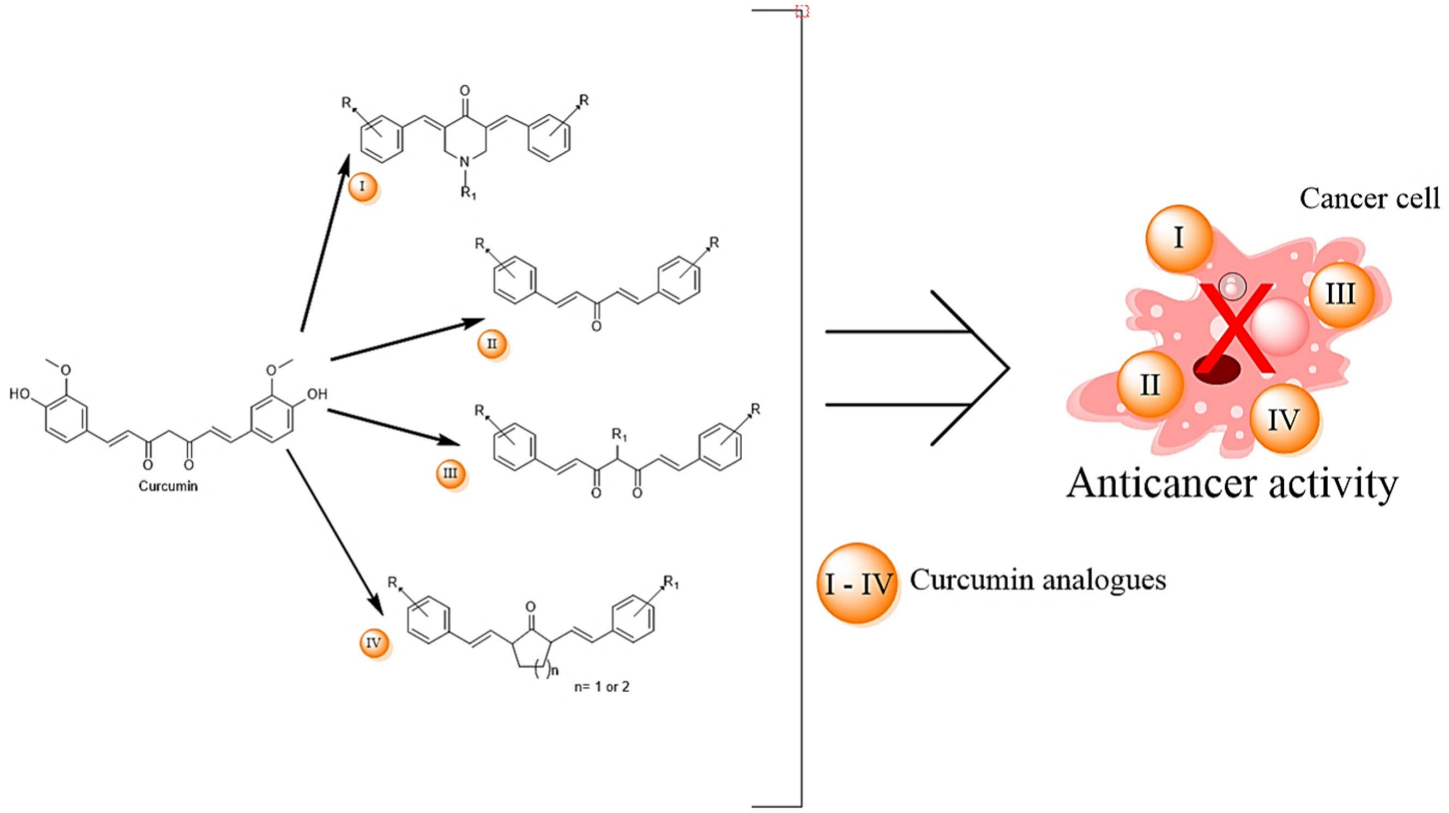
Curcumin analogues and their use in cancer treatment.

Jiang et al. identified the antitumor constituents in curcuminoids from *C. longa* (Figure 6) on He La cells, demonstrating a significant correlation between curcuminoids and antitumor activity. The inhibitory role of CUR in lipolysis was investigated in 3 T3-L1 adipocytes, revealing its potential to attenuate TNF-α-mediated lipolysis. This antilipolytic effect could underlie CUR’s ability to reduce plasma free fatty acid levels and improve insulin sensitivity (20).

CUR emerged as a potent tight binding inhibitor of human carbonyl reductase 1 (CBR1), inhibiting daunorubicinol formation. This inhibition could enhance the therapeutic effectiveness of daunorubicin by preventing heart tissue damage (21).

Yodkeeree et al. (22) conducted a study to compare the impact of CUR, DMC, and BDMC on the expression levels of urokinase plasminogen activator, metalloproteinases (MMPs), membrane type 1 (MT1-MMP), tissue inhibitor of MMPs, and the *in vitro* invasiveness of human fibrosarcoma cells. The order of potency in inhibiting cancer cell invasion was found to be BDMC > DMC > CUR. Zymography analysis revealed that, in a dose-dependent manner, CUR, DMC, and BDMC significantly reduced urokinase plasminogen activator and active MMPs from the cells. Notably, BDMC and DMC exhibited greater potency in this regard compared to CUR. All three forms of curcuminoids significantly inhibited collagenase and MMPs. DMC and BDMC demonstrated higher antimetastatic efficacy than CUR, attributable to their differential down-regulation of extracellular matrix (ECM) degradation enzymes (23).

The administration of DMC resulted in the inhibition of nuclear factor-kappa B’s DNA binding activity, a factor that orchestrates the expression of MMPs, urokinase plasminogen, intercellular adhesion molecule-1, and chemokine receptor 4. The anti-invasive effect of DMC appears to be primarily mediated through the modulation of the expression of proteins associated with invasion, potentially by targeting nuclear factor-kappa B in MDA-MB-231 cells (24). Moreover, curcuminoids-mediated photodynamic therapy (PDT) exhibited a substantial suppression of cell viability in breast cancer cell lines, with DMC-PDT demonstrating the most pronounced anti-proliferative effect. The potential of DMC as a novel photosensitizer in PDT for cancer treatment was substantiated by its ability to reverse cell viability, reduce LC3 conversion, and inhibit PARP cleavage, all of which were attenuated by pre-treatment with a singlet oxygen scavenger or JNK inhibitor in the context of DMC-PDT. Notably, DMC-PDT displayed superior efficacy compared to DMC alone in curtailing cell viability in breast cancer cell lines, suggesting its promising role as a potential photosensitizer in cancer therapy (25, 26).

The metabolic profile of *Rhizoma paridis* saponins combined with turmeric intervention in H22 hepatocarcinoma mice showed promising anticancer effects by suppressing levels of amino acids, lipid compounds, and carbohydrates in tumor tissues (27).

In a research investigation focused on the monocarbonyl analogue of B63, derived through chemical modifications of curcumin’s structure, this compound demonstrated a heightened antiproliferative impact compared to curcumin specifically on colon cancer cells. Furthermore, utilizing a lower dosage of B63 (50 mg/kg B63 versus 100 mg/kg curcumin) still resulted in the suppression of tumor growth, akin to the effects observed with curcumin (28).

### 3.2 Radioprotective effects

Curcuminoids, as potent antioxidant polyphenols, exhibit radiomodulatory properties by conferring radioprotection to non-cancerous cells while sensitizing tumor cells to radiation. In a study conducted by Lopez-Jornet et al. (29) the potential protective effects of lycopene and CUR on the parotid glands of female Sprague Dawley rats during radiotherapy were explored. Morphological and histopathological analyses revealed reduced cell necrosis in the CUR-treated group compared to other groups. Pre-administration of lycopene and CUR 24 h before irradiation contributed to mitigating structural damage to the salivary glands. Sebastià et al. (30) reported a dual action of polyphenols present in CUR, manifesting as both radioprotective and radiosensitive effects. The observed radiosensitization was attributed to compromised G2-checkpoint functionality, diminishing its capacity to effectively halt damaged cells in the G2-phase and resulting in a significant increase in radiation-induced chromatid breaks. The simultaneous dual-mode action of these polyphenols suggests that the overall net effect—whether radioprotective or radiosensitizing—depends on the cell-cycle status of the cells at the time of irradiation (31, 32).

Belcaro et al. (33) conducted a clinical investigation evaluating a specialized lecithin delivery system of CUR (Meriva) in 160 cancer patients undergoing chemotherapy and radiotherapy. The study findings led the authors to conclude that the formulated CUR has the potential to reduce the pain-related side effects associated with cancer therapy (33).

In another clinical trial involving 30 breast cancer patients, the protective effects of Curcumin C3 Complex® (6 g/day) against radiodermatitis were assessed. Parameters such as moist desquamation, pain level, redness, and severity of radiation dermatitis were measured. The curcumin group exhibited a significant reduction in moist desquamation and the severity of radiation dermatitis compared to the placebo group (35). Another study investigated the effectiveness of Vicco® turmeric cream (Vicco Laboratories, Parel, India), containing sandalwood and turmeric oil, in alleviating radiodermatitis induced by radiotherapy in 50 patients with head and neck cancer. The cream was applied daily (five times a day) from the first day and continued until 2 weeks after treatment completion. Acute skin reactions were monitored twice a week. Results indicated a notable decrease in dermatitis grades among patients using Vicco® turmeric cream at all evaluated time points (36, 37).

### 3.3 Anti-inflammatory effects

Inflammation represents a fundamental and noteworthy defensive mechanism employed by organisms in response to tissue damage. This reaction is elicited by various factors, including ischemic injury resulting from insufficient blood supply to a tissue or organ, physical trauma, exposure to toxins, infection, or other forms of trauma (38). It is imperative to effectively curtail the inflammatory response once its necessity diminishes to prevent undesirable tissue damages and cellular destruction, potentially leading to chronic inflammation (39).

The inflammatory process involves the participation of leukocytes or inflammatory cells, namely neutrophils, lymphocytes, and macrophages. Subsequent to the inflammatory cascade, leukocytes release specific elements such as eicosanoids, vasoactive peptides and amines, cytokines, and acute-phase proteins. These factors act in concert to mediate the inflammatory procedure, thereby averting further tissue damage and ultimately facilitating the healing and restoration of tissue function (40).

Curcumin, a component extensively utilized in Eastern medicine, has demonstrated therapeutic efficacy in treating various chronic diseases and inflammatory disorders, including airborne diseases. Attributed to its phenolic composition, curcumin exhibits antioxidant properties, preventing apoptosis by promoting the growth of inhibited cells. Turmeric, containing curcumin, enhances safety in food by preventing peroxide formation and surpasses vitamin E in effectively preventing lipid oxidation. Components extracted from *Curcuma longa* display significant antioxidant effects, playing a crucial role in preventing lipid oxidation (41, 42).

**Figure 7:**
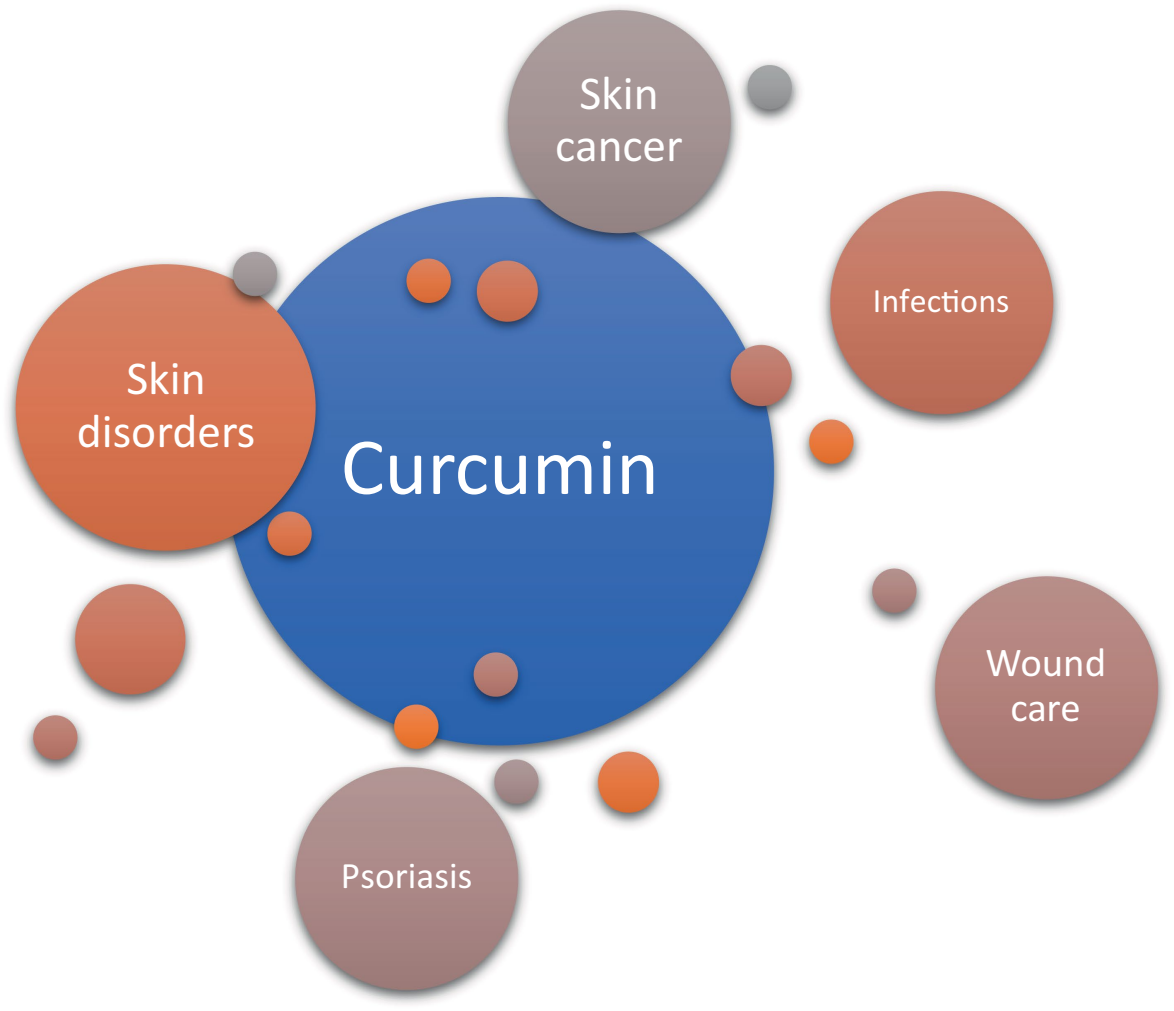
The effects of applying curcumin products to skin lesions and pathologies.

Traditionally, turmeric has been topically applied for skin diseases, insect bites, and chickenpox in India, serving as an alternative medical support for wound healing (Figure 7). Curcumin treatment accelerates wound contraction, increases fibronectin and collagen expression in myofibroblasts, and enhances granulation tissue formation and neovascularization in mouse-wound models with diabetes and hydrocortisone (43). Curcumin reduces injuries induced by hydrogen peroxide in yellow keratinocytes and fibroblasts, significantly decreasing wound healing time. In mouse models, curcumin exhibits antiulcer effects, reducing lipid peroxidation and protein oxidation, and promoting re-epithelialization to reverse gastric epithelial cell damage (44).

Numerous common skin disorders are associated with the dysregulation of the inflammatory response. Curcumin has demonstrated the ability to down-regulate various inflammatory targets, including lipoxygenase, cyclooxygenase-2, and inducible nitric oxide synthase. Additionally, it acts as an inhibitor of several inflammatory cytokines, such as TNF-α, interleukin-1, −2, −6, −8, and − 12 (45). The transcription factor nuclear factor kappa B (NF-κB), which governs cyclooxygenase-2 and inducible nitric oxide synthase and regulates cellular proliferation, is proposed to be suppressed by curcumin (46). TNF-α, implicated in psoriasis and atopic dermatitis, triggers proinflammatory cytokines and activates NF-κB (47). Hence, the potential reduction of NF-κB by curcumin could contribute to its therapeutic efficacy in managing inflammatory skin diseases (48). Mohanty et al. applied a curcumin-loaded oleic acid-based polymeric bandage (COP) topically on the backs of wounded rats and observed a downregulation in the expression of various kinases in the PI3K/AKT/NF-κB pathway. The application of the COP bandage resulted in the downregulation of P13K and pAKT kinases, leading to reduced activation of the NF-κB gene and decreased inflammation. Additionally, an upregulation in I-κB-α protein, which inhibits the NF-κB pathway, was observed. Therefore, Mohanty et al. demonstrated that curcumin effectively reduces inflammation at wounded sites by modulating the NF-κB pathway (49). In contrast to Mohanty’s findings, an *in vivo* study reported an increase in inflammatory cell infiltration in burn wounds on rats treated with curcumin compared to untreated groups (50). However, the study did not specify the type of inflammatory cells measured, necessitating further investigations to elucidate the proinflammatory effects of curcumin on wounds. Interestingly, curcumin was also found to enhance nitric oxide (NO) production in excision wounds of mice exposed to gamma radiation (51). Increased NO production has been shown to promote wound healing in patients by enhancing inflammation (35). Although Jagetia and Rajanikant proposed that the increase in NO contributed to improved wound healing with curcumin treatment, the majority of studies provide evidence that curcumin indeed reduces inflammation. By mitigating the inflammatory response, damaged skin can more efficiently progress to later stages of healing, such as proliferation and remodeling. Uncontrolled and prolonged inflammation may delay these subsequent stages and impede the overall wound healing process (52). Despite its potent modulative effects on wound healing, curcumin faces challenges related to low bioavailability, rapid metabolism, inadequate solubility, and sensitivity. Exploring new formulations, such as nanoparticles, is crucial to overcoming these limitations and harnessing the full potential of curcumin (53).

Curcumin’s also influence extends to inhibiting platelet production, removing mitogens that stimulate the rapid growth of mononuclear blood cells, and partially inhibiting the protein kinase enzyme (54). Given the well-established role of oxidative stress in the pathogenesis of various diseases (e.g., myocardial ischemia, ischemia–reperfusion, bleeding, shock, nerve cell damage, and cancer), curcumin’s anti-inflammatory and antioxidant properties are substantiated. It eliminates various forms of reactive oxygen species (ROS), including hydroxyl radicals and nitrogen dioxide radicals. The antioxidant capacity of curcuminoids has been reported to be equivalent to that of ascorbic acid (55).

The inflammation and the oxidative stress and its associated alterations in neuroplasticity play pivotal roles in the development of this neurodevelopmental disorder. Recent studies have suggested a potential role for curcumin in the treatment of depression and bipolar disorder. Adding curcumin to antidepressant drugs has demonstrated significant reductions in depressive symptoms compared to a placebo supplement. Furthermore, a recent meta-analysis has provided support for the effectiveness of adjunctive curcumin in managing depression and anxiety disorders. Importantly, curcumin has been shown to be well-tolerated and safe in various randomized clinical trials involving humans (56).

**Figure 8:**
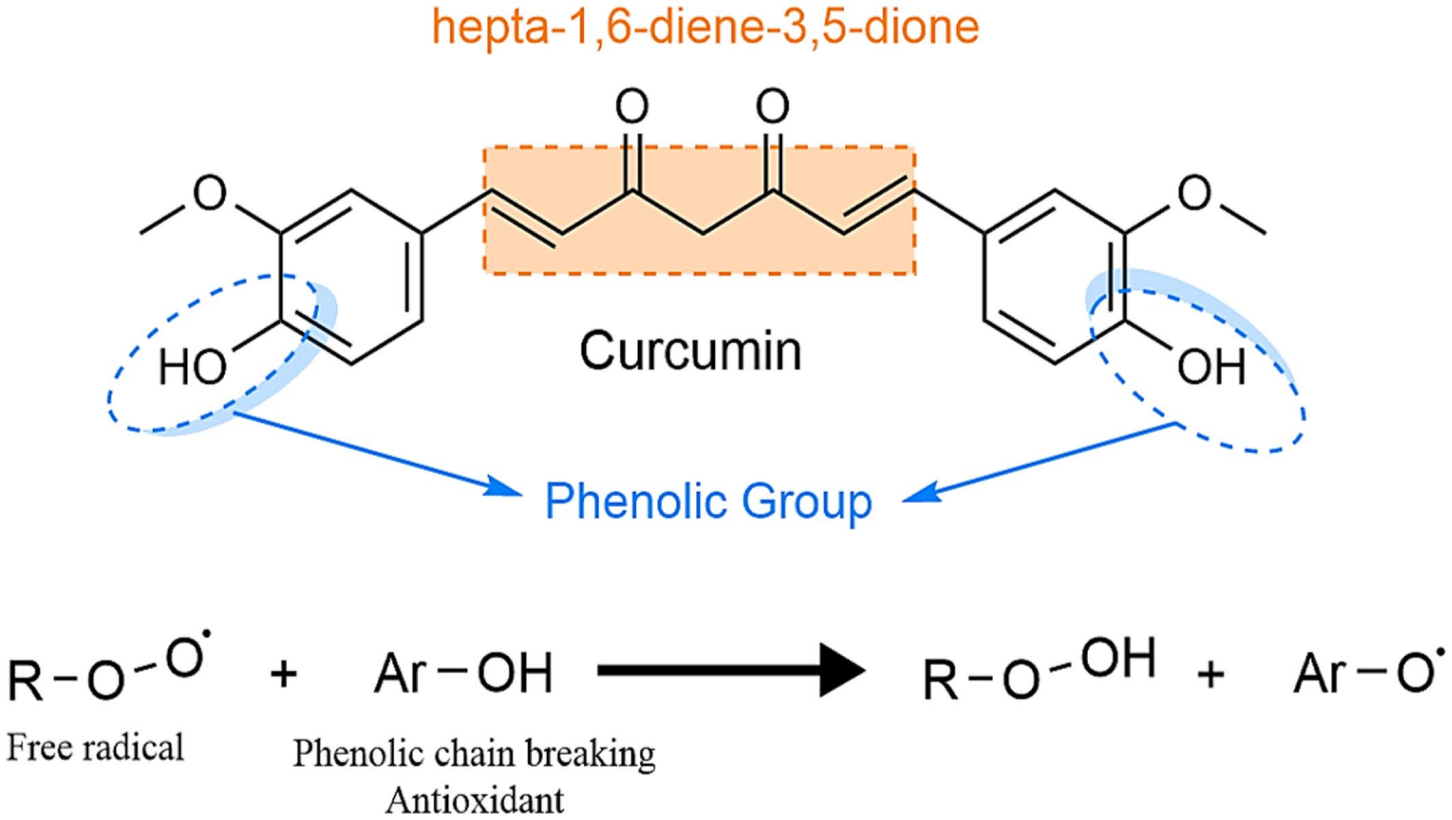
The antioxidant action of curcumin.

Functioning as a potent hydroxyl radical scavenger and superoxide radical capturer (Figure 8), curcumin protects DNA from oxidative injury by retaining free radicals. Following oral intake, it undergoes hydrogenation in the intestines, transforming into tetrahydrocurcumin, and is subsequently absorbed, distributed into the blood and tissues, and excreted in the bile. Curcumin supplementation has been shown to reduce muscle damage induced by eccentric exercise in rats (57).

Multifaceted anticancer effects of turmeric encompasses various aspects, including the modulation of key cellular pathways, the impact on specific cancer cell lines, the inhibition of metastasis, and the exploration of potential therapeutic applications. Curcumin, a primary constituent of turmeric, has exhibited remarkable efficacy throughout multiple stages of cancer progression. Its inhibitory effects extend to the transformation, initiation, development, and invasion of tumors, as well as angiogenesis and metastasis. This broad spectrum of action positions curcuminoids as potent suppressors of tumor cell growth.

#### 3.3.1 Mechanistic insights

The discussion delves into the intricate molecular mechanisms underlying curcuminoids’ anticancer effects. The modulation of crucial cellular pathways, including the cell proliferation, cell survival, caspase activation, tumor suppressor, and death receptor pathways, elucidates the diverse strategies employed by curcuminoids in targeting cancer cells. These pathways involve key regulators such as cyclin D1, c-myc, Bcl-2, Bcl-xL, caspases, p53, p21, DR4, and DR5 (58).

#### 3.3.2 Cell-line specific effects

Studies on specific cancer cell lines, such as MCF-7 human breast tumor cells, highlight the differential potency of curcuminoids. For instance, DMC demonstrates superior inhibitory effects compared to CUR and BDMC, emphasizing the importance of structural elements like phenolic hydroxyl groups, methoxyl groups, and the diketone moiety.

### 3.4 Potential therapeutic applications

The research extends its focus to potential therapeutic applications. In leukemia treatment, pure CUR shows promise in modulating the expression of the WT1 gene, indicating its potential utility in leukemia therapy. Furthermore, the exploration of curcuminoids in lipolysis inhibition suggests a potential avenue for reducing plasma free fatty acid levels and improving insulin sensitivity. The order of potency in inhibiting cancer cell invasion is identified as BDMC > DMC > CUR, and their ability to significantly reduce urokinase plasminogen activator and active MMPs underscores their potential in inhibiting invasion and metastasis (59). The discussion introduces the application of curcuminoids in photodynamic therapy (PDT), revealing their substantial suppression of cell viability in breast cancer cell lines. The heightened anti-proliferative effect observed with DMC-PDT, coupled with its potential as a novel photosensitizer, suggests a promising avenue for cancer therapy. Additional studies explore the metabolic profile of curcuminoids in combination with other agents, showcasing their anticancer effects by suppressing levels of amino acids, lipid compounds, and carbohydrates in tumor tissues. The discussion also touches upon the enhanced antiproliferative impact of a monocarbonyl analogue of B63 compared to curcumin, specifically in colon cancer cells (60).

Regarding radiomodulatory properties of curcuminoids, there are a collection of studies exploring turmeric bioactive compounds particularly curcumin, and their potential applications in the context of cancer therapy. These studies both preclinical and clinical investigations, shedding light on the multifaceted effects of curcuminoids in the presence of radiation.

Studies show a dual nature of curcuminoids, acting as both radioprotectors for normal cells and radiosensitizers for tumor cells. The study by Lopez-Jornet et al. (29) on female Sprague Dawley rats during radiotherapy elucidates the potential protective effects of curcumin on parotid glands. Morphological and histopathological analyses revealed reduced cell necrosis in the CUR-treated group, indicating a radioprotective effect. Furthermore, the pre-administration of lycopene and CUR contributed to mitigating structural damage to the salivary glands. (30) reported a dual action of polyphenols in CUR, manifesting both radioprotective and radiosensitive effects. The radiosensitization was attributed to compromised G2-checkpoint functionality, leading to increased radiation-induced chromatid breaks (61).

Inflammation, oxidative stress, and neuroplasticity-related changes play key roles in the development of this neurodevelopmental disorder. Recent studies have suggested a potential role for curcumin in the treatment of depression and bipolar disorder. Adding curcumin to antidepressant medications has shown significant reductions in depressive symptoms compared to a placebo supplement (62).

**Figure 9:**
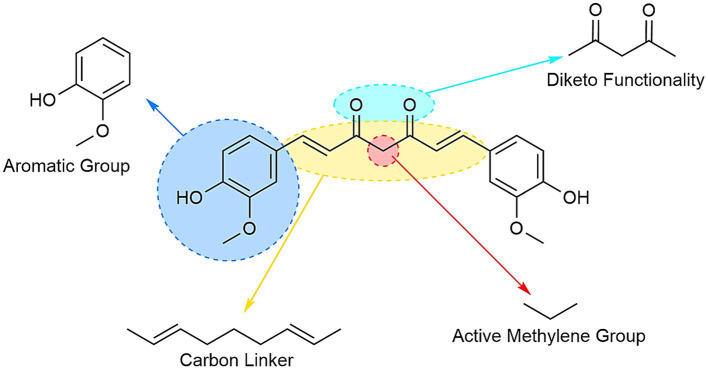
The building blocks of the curcumin molecule and its multifaceted uses.

Curcumin (Figure 9) has the complexity of a long carbon chain that bonds at the ends with two benzenes. Along this chain there can be found both carbonyl and hydroxyl oxygens which provide curcumin its specific character, besides these the double bonds also have a role in the bioavailability and polymerization of multiple molecules and the formation of bonds with other diverse componds.

The diketo moiety can also act as a potent metal chelator, coordinationg metal ions and forming with them a complex salt. The phenolic hydroxyl groups also act as potent antioxidants by donating their hydrogens to free radicals, minimizing the formation of reactive oxygen species and inhibiting oxidative stress.

In summary, the cumulative evidence presented in the text underscores the multifaceted and promising anticancer properties of curcuminoids. From elucidating molecular mechanisms to exploring specific applications, the diverse range of studies contributes valuable insights into the potential of curcuminoids as effective agents in cancer therapy.

This review highlights curcumin’s inhibitory effects on various stages of cancer progression, including transformation, initiation, development, invasion, angiogenesis, and metastasis. This broad spectrum of action positions curcuminoids as potent suppressors of tumor cell growth. The involvement of key regulators such as cyclin D1, c-myc, Bcl-2, caspases, p53, p21, DR4, and DR5 is explored, providing a mechanistic understanding of the diverse strategies employed by curcuminoids in targeting cancer cells. Potential therapeutic applications, including leukemia treatment, lipolysis inhibition, and photodynamic therapy, are explored, showcasing the versatility of curcuminoids in diverse cancer-related contexts (63, 64).

In the context of cancer therapy, we find a dual nature of curcuminoids, acting as both radioprotectors for normal cells and radiosensitizers for tumor cells. Insights from preclinical and clinical investigations shed light on the potential of curcuminoids in minimizing radiation-induced damage to normal tissues while enhancing the sensitivity of tumor cells to radiation. The findings collectively suggest that curcuminoids have promising applications in cancer therapy, acting both as protectants for healthy cells and sensitizers for cancer cells. Despite valuable insights, the article acknowledges potential limitations and calls for future research to standardize methodologies, explore long-term effects, and elucidate molecular mechanisms. Randomized controlled trials are proposed to strengthen the scientific basis for integrating curcuminoids into cancer treatment regimens (65, 66).

## 4 Conclusion

In this review we find the role of curcumin in modulating inflammatory responses and promoting wound healing. Through its antioxidant properties and the down-regulation of inflammatory targets, curcumin emerges as a promising agent in managing inflammatory skin diseases. Discrepancies in findings prompt further investigations, while the acknowledgment of challenges underscores the need for innovative formulations like nanoparticles to maximize curcumin’s therapeutic potential in wound healing.

The present article provides a comprehensive overview of curcumin’s multifaceted roles in cancer therapy, inflammation, and wound healing. The diverse applications of curcuminoids in cancer treatment, coupled with their immunomodulatory properties, present exciting prospects for future research and clinical applications. The nuanced understanding of curcumin’s mechanisms of action contributes to its potential integration into mainstream cancer therapies and wound care, offering a natural and versatile approach to disease management.

Novel aspects underlined in the review paper are related to the versatility of curcuminoids in cancer-related contexts, showcasing potential therapeutic applications beyond traditional chemotherapy. In the realm of cancer therapy, we uncover a dualistic property of curcuminoids, wherein they serve as both radioprotective agents for healthy cells and radiosensitizing agents for tumor cells. This distinctive attribute carries implications for mitigating radiation-induced harm to normal tissues while concurrently augmenting the susceptibility of tumor cells to radiation therapy.

While acknowledging the promising therapeutic potential of curcuminoids, we have uncovered potential limitations such as the low bioavailabiliy of the molecule and its fast metabolism, althought these present as obstacles, they can be used to our advantage for making different curcumin formulations that are easily metabolized and do not leave traces behind. Besides these, the bioavailabily of curcumin can be potentially increased by combining it with different bioavailable molecules. Curcumin can also be used as a chelator for different metal ions, for adminestering or removing them from the body, this property seems to be useful in different supplements.

In summary, our review has uncovered the multifaceted potential of curcumin both as an immunomodulator, as a radioprotective, anticancer medication and so much more. Curcumin boasts multiple benefits and presents itself as an interesting subject for future research.

## Author contributions

MC: Writing – original draft, Writing – review & editing. IIL: Writing – original draft, Writing – review & editing. CrG: Writing – original draft, Writing – review & editing. AS: Writing – original draft, Writing – review & editing. LDD: Writing – original draft, Conceptualization, Software. BDS: Writing – original draft, Writing – review & editing. DD: Writing – original draft, Writing – review & editing. GC: Writing – original draft, Writing – review & editing. ERBG: Writing – original draft, Writing – review & editing. CaG: Writing – original draft, Writing – review & editing. MB: Writing – original draft, Writing – review & editing.
